# Life-threatening aortic thrombosis in a trauma patient homozygous for factor V Leiden mutation: Case report

**DOI:** 10.1186/1477-9560-9-8

**Published:** 2011-05-09

**Authors:** Iraklis Tsangaris, Georgios Tsaknis, Argirios Tsantes, Petros Kopterides, Apostolos Armaganidis

**Affiliations:** 1 2nd Critical Care Department, Athens University School of Medicine - "Attiko" University Hospital, 1 Rimini Str., Haidari - Athens, 12462, Greece; 2 Laboratory of Hematology & Blood Bank Unit, Athens University School of Medicine - "Attiko" University Hospital, 1 Rimini Str., Haidari - Athens, 12462, Greece

## Abstract

We report a case of near fatal aortic thrombosis in a trauma patient homozygous for mutation of Factor V Leiden. He responded well to vascular surgery and intensive care unit management and was discharged successfully from the hospital one month later.

## Background

Thrombophilia may be described as a congenital or an acquired tendency to arterial and venous thrombosis. Mutation of Factor V Leiden (FVL) appears as a major predisposing factor for venous thromboembolism and has a high prevalence in the Caucasian population [1]. The relative risk (RR) for the first incident of venous thrombosis and the annual incidence (AI) values associated with this mutation are 7.0 and 0.06%, respectively, in heterozygotes. For homozygotes, RR and AI values are projected to be as high as 80 and 0.5-1.0%, respectively [2,3].

The lifetime probability of developing thrombosis is 2.2 for carriers of the FVL mutation, which is quite low compared to carriers of antithrombin deficiency (8.1), protein S deficiency (8.5) and protein C deficiency (7.3) [4]. There is still debate regarding the utility of lifetime anticoagulation in patients with FVL, with one study randomizing patients on warfarin versus placebo showing a risk value reduction in recurrent venous thromboembolism of 76-80% in the warfarin group. According to the investigators, anticoagulation therapy should exceed a 3-month period after the first venous thromboembolic episode with conventional international normalized ratio (INR) target between 2 and 3 [5,6]. Carriage of FVL mutation is highly linked to venous but not arterial thrombosis; however, there is growing evidence linking this mutation to an increased risk for arterial involvement, specifically for myocardial infarction [7-9]. We herein present a rare case of aortic thrombosis in a trauma patient that was found to be homozygous for FVL mutation.

## Case presentation

A 32-year-old male, presented to the ER of an outside hospital complaining about numbness in both legs after being involved in a street fight 3 days ago. He was a non-smoker with non-significant previous medical history. Physical examination was unrevealing and vital signs were normal. Radiologic evaluation revealed only a fracture of the 10^th ^left rib and the patient was discharged with instructions for bed rest.

**Figure 1:**
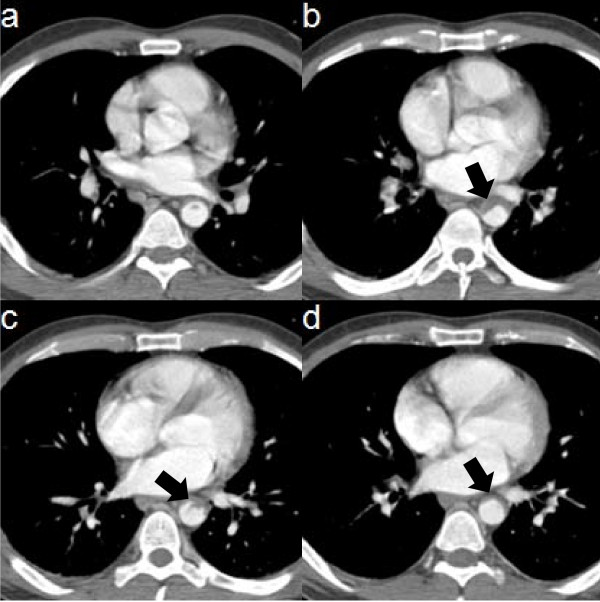
**CT images demonstrating the occlusive thrombus in the descending thoracic aorta (black arrows)**.

In the next 24 hours he gradually developed abdominal pain and complained about excessively numb and cold legs with normal urination and defecation. By the time he presented to the ER of the same hospital, he could not move his legs. On physical examination, there were no palpable pulses in all arterial sites of both lower extremities. An emergent CT angiography was performed revealing a large (5.8 cm length and 0.8 cm width) thrombus in the descending aorta, causing partial occlusion of its lumen (Figure 1), as well as an infra-renal thrombus extending into both iliac branches, causing complete lumen occlusion. He was started on intravenous drip of unfractionated heparin, clopidogrel and warfarin and a few hours later he was transferred to our department for further management.

A successful surgical recanalization was immediately performed by the vascular surgeons and postoperatively the patient was transferred intubated to our intensive care unit. He subsequently developed rhabdomyolysis with a peak creatine kinase (CK) and myoglobin levels of 130,000 U/L (with CK-MB value of 15,000 U/L) and 3,000, respectively. The LDH level was 6,545 U/L and liver function tests revealed AST and ALT levels of 1,297 and 340 U/L, respectively. He also developed acute renal failure (ARF), hyperkalemia and severe tibial compartment syndrome of the left leg necessitating fasciotomies.

Taking into account the patient's young age, complicated clinical course and laboratory hypercoagulative profile (PLTs were 625,000/ml and fibrinogen was 663 mg/dL), as well as the fact that he had no other clinical or imaging evidence of venous thrombosis, we decided to perform a thrombophilia work-up. The rapid and life-threatening course of the disease had understandably forced the referring physicians from the outside hospital to initiate aggressive anticoagulation therapy. This did not allow us to perform antithrombin III, protein C and S testing. However, the patient was found to carry a homozygous mutation for FVL and also heterozygous mutations for Factor XIII and beta-FIBR. Of interest, his family history was non-significant for thromboses. When renal function improved, we switched from unfractionated heparin to low-molecular weight heparin and administered warfarin aiming at a therapeutic target INR between 2 and 3.

During the rest of his ICU hospitalization he remained hemodynamically stable, his ARF responded well to continuous veno-venous hemodiafiltration and was finally extubated on day 15. The patient was transferred to the Vascular Surgery Department for further management and discharged from the hospital a month later.

## Discussion

Cases of heterozygous FVL mutation causing arterial disease are rare and have been described mostly in neonates [10-12]. Other cases of aortic arch and peripheral arterial thrombosis have also been reported in adults [13,14]. The synergistic effects of FVL mutation and other prothrombotic conditions such as homocystinemia, protein C and S deficiency, oral contraceptives and pregnancy have been also previously described [15].

There are cases of post-traumatic arterial thrombosis of several vascular sites (femoral, renal, cranial, abdominal), not necessarily associated with prothrombotic conditions [16-18]. The incidence of abdominal aortic trauma resulting from blunt force is only around 5% and interestingly the majority of thrombi occur in abdominal aorta [19].

A Medline search triggered by the current case report (literature review performed on April 2, 2011) and using the search terms "*abdominal aortic thrombosis*" AND "*trauma*" revealed 15 case reports and 2 case series. Thrombotic episodes were mainly associated with major blunt trauma in automobile accidents (predominantly during childhood), with no clear predisposing thrombotic factors. The low reported number of similar cases does not allow us to safely determine whether the finding of a hypercoagulable genetic profile in a trauma patient experiencing a thrombotic episode is causally linked or merely co-incidental. However, we support the notion that a thrombophilia work-up might be a prudent choice when extensive thromboses are observed in young trauma patients suffering low-impact traumatic injuries.

## Conclusions

Homozygous mutation of FVL as well as the arterial site of the first thrombotic event are rare incidents. Although arterial thrombosis occurs commonly on sites of previous vascular pathology (i.e. an atherosclerotic plaque), recent clinical evidence suggests that in cases of extensive arterial thrombosis, a thrombophilia work-up may be required. Aggressive surgical management may be needed if the arterial incident is severe while the lifetime use of warfarin prophylaxis is still debated.

## Consent

Written informed consent was obtained from the patient for publication of this case report and any accompanying images. A copy of the written consent is available for review by the Editor-in-Chief of this journal.

## Competing interests

The authors declare that they have no competing interests.

## Authors' contributions

TI and PK participated in patient management and data collection, TG participated in patient management, data collection and drafted the manuscript, TA carried out the thrombophilia work-up and AA participated in patient management, supervised and approved the manuscript. All authors read and approved the final manuscript.
